# *Pseudomonas aeruginosa* injection for the treatment of chyle fistula following neck dissection in thyroid cancer

**DOI:** 10.3389/fendo.2025.1648802

**Published:** 2025-11-27

**Authors:** Defeng Chen, Zhen Zhu, Qiufeng Jin, Junhui Yuan, Xu Zhang, Qigen Fang

**Affiliations:** 1 Department of Head Neck and Thyroid, The Affiliated Cancer Hospital of Zhengzhou University and Henan Cancer Hospital, Zhengzhou, China; 2 Department of Radiology, The Affiliated Cancer Hospital of Zhengzhou University and Henan Cancer Hospital, Zhengzhou, China

**Keywords:** chyle fistula, *Pseudomonas aeruginosa*, neck dissection, thyroid surgery, octreotide

## Abstract

**Objective:**

To present our experiences with *Pseudomonas aeruginosa* injection (PAI) for managing chyle fistula (CF) following thyroid surgery, with a focus on its efficacy and safety.

**Methods:**

We conducted a retrospective, single-center study enrolling patients with CF. Patients were categorized into low- (~500 mL/d), moderate- (500-1000 mL/d), and high-output (>1000 mL/d) groups. The primary outcomes were the efficacy and safety of PAI.

**Results:**

A total of 95 patients were included. Among them, eight with low-output CF, five with moderate-output CF, and 15 with high-output CF underwent PAI. Following PAI, a significant reduction in drainage volume was observed, resulting in the removal of 92.9% of drainage tubes by the third day post-treatment. Fever and pain of varying intensity were common adverse effects immediately after PAI. However, by the third day, no patients had a fever, and no moderate or severe pain was reported. Based on our findings, we propose that initial CF management should be stratified by drainage output. The majority of cases were effectively resolved using PAI and other non-surgical interventions, with reoperation reserved only for when conservative treatments failed.

**Conclusion:**

PAI proved effective in resolving CF after unsuccessful prior non-surgical interventions following thyroid surgery, with minimal complications. Management of CF requires a tiered approach based on output levels.

## Introduction

Thyroid cancer stands as the most prevalent neoplasm among all endocrine malignancies, with lymph node metastasis occurring in up to 60% of cases; notably, lateral neck metastasis presents in approximately 30% of patients (1). In such instances, total thyroidectomy accompanied by lateral neck dissection encompassing levels II to IV/V is imperative. Nonetheless, even when performed by seasoned specialists, postoperative complications cannot be entirely averted.

Chyle fistula (CF), although rare, poses a potentially life-threatening complication following neck dissection. If not managed promptly, it can lead to dire repercussions including malnutrition, fluid and electrolyte imbalances, immune system dysfunction, and skin necrosis (2). Conservative management, which typically involves dietary restrictions, parenteral nutrition, pressure dressings, and somatostatin application, is generally the initial treatment approach; however, its efficacy is often limited and typically necessitates prolonged hospitalization (3–5).

*Pseudomonas aeruginosa* has been documented to possess fimbriae that can stimulate a Th1-type immune response, thereby facilitating the maturation and migration of macrophages, natural killer cells, monocytes, and dendritic cells (6). The injection of *Pseudomonas aeruginosa* (PAI) has been utilized in the treatment of malignant pleural effusion, pericardial effusion, and recurrent seromas following axillary lymphadenectomy (7). Furthermore, pioneering research from a renowned center in China (8–12) has elucidated the safety and feasibility of employing PAI in the management of CF and in reducing drainage volumes post-thyroid surgery. However, these findings have yet to be validated in other institutions, and are limited by their sample size.

Thus, our objective is to delineate our experiences with PAI in the treatment of CF subsequent to thyroid surgery, focusing on its efficacy and safety.

## Patients and methods

### Ethical approval

This study was approved by the Institutional Research Committee of Henan Cancer Hospital. Written informed consent for participation in medical research was obtained from all patients prior to the initiation of treatment. All procedures were conducted in strict accordance with relevant guidelines and regulations.

### Study design

We conducted a retrospective analysis of prospectively collected data. Between January 2015 and April 2024, adult patients diagnosed with CF following thyroid cancer surgery with neck dissection were included. The sole inclusion criterion was a confirmed diagnosis of CF, based on milky drainage with a triglyceride concentration >100 mg/dL. Exclusion criteria included: significant immunocompromised states (HIV/AIDS, ongoing chemotherapy, or chronic high-dose steroid use), active systemic infections, known hypersensitivity to the PAI preparation, and inability to provide informed consent. There were no specific exclusion criteria based on BMI or age. All enrolled patients were medically fit to undergo the proposed treatment. Demographic, pathologic, treatment, and follow-up data were recorded.

### Study variables

CF was suspected based on an increase in daily drainage volume with a milky or ivory-white appearance and was confirmed by a triglyceride concentration exceeding 100 mg/dL. CF was classified into three groups: high-output (>1000 mL/24h), moderate-output (500-1000 mL/24h), and low-output (≤500 mL/24h). Fever was defined as a maximum body temperature exceeding 38.5 °C on any given day. One drain was placed in the thyroid region, with an additional drain placed for each lateral neck region dissected. The criteria for drain removal were a 24-hour drainage volume of ≤20 mL with no evidence of bleeding or persistent chyle leak.

The primary outcome was the efficacy of PAI, assessed through drainage volume and duration, surgical site infection rates, and reoperation rates. The secondary outcome was the safety of PAI, evaluated by monitoring adverse events, including body temperature fluctuations and pain levels. Body temperature was recorded as the highest value each day. Pain was quantified using a visual analog scale (VAS, where 0 = no pain and 10 = extreme pain). Pain scores were patient-reported during routine clinical assessments and documented in the electronic medical records by nursing staff.

### Treatment

All surgical procedures were performed by the same surgical team via an open neck incision. The lateral compartment was dissected superiorly to the posterior belly of the digastric muscle, inferiorly to the subclavian vein, and laterally to the anterior border of the trapezius muscle. A low-fat or fat-free diet was recommended for all patients postoperatively.

The PAI preparation (Beijing Wanter Biopharmaceutical Company, Beijing, China; National Drug Approval No. S19990063) was derived from the CMCC(B)10104 strain. The preparation involves culturing *Pseudomonas aeruginosa* followed by inactivation using heat treatment and formaldehyde, resulting in a non-viable suspension that retains immunostimulatory properties while being devoid of virulence. This preparation is approved by the National Medical Products Administration of China for clinical use in managing malignant effusions and has been utilized off-label for chyle fistula at our institution.

During PAI therapy, a concentrated 2 mL dose was injected through the drainage tube, after which the distal end of the tube was clamped for a minimum of 30 minutes. No prophylactic or therapeutic antibiotics were administered in conjunction with PAI treatment.

### Statistical analysis

Primary and secondary outcome variables are presented descriptively. Given the exploratory nature of this study and the multiple pairwise comparisons among the three output groups for continuous variables, we applied the Bonferroni correction to control the family-wise error rate and reduce the likelihood of Type I errors. Due to the relatively modest subgroup sample sizes and the primary descriptive focus of this analysis, we prioritized clarity in reporting group differences over developing multivariate predictive models; therefore, regression analysis was not performed. All statistical analyses were two-tailed, conducted using R version 3.4.4, with a significance threshold set at P < 0.05.

## Results

### Baseline data

Between January 2015 and April 2024, 105 patients were identified with CF following thyroid surgery. After applying the exclusion criteria, 10 patients were excluded for the following reasons: immunocompromised status (n=4), active systemic infection (n=3), known hypersensitivity to PAI (n=1), and incomplete medical records (n=2). The final analysis included 95 patients with a mean age of 45 ± 14 years; the cohort consisted of 37 males (38.9%) and 58 females (61.1%). Forty-four patients (46.3%) had a normal BMI (18.5-22.9), while 51 (53.7%) had a BMI greater than 23. The distribution of tumor stages was as follows: T1 in 23 patients (24.2%), T2 in 29 patients (30.5%), T3 in 28 patients (29.5%), and T4 in 15 patients (15.8%). All patients underwent total thyroidectomy. This was accompanied by unilateral neck dissection (levels II-V) in 63 patients (66.3%) and bilateral neck dissection (levels II-V) in 32 patients (33.7%). Pathological diagnoses included papillary thyroid carcinoma in 73 patients (76.8%) and medullary thyroid carcinoma in 22 patients (23.2%).

### Before PAI

CF was established one day postoperatively in 15 patients (15.8%), two days postoperatively in 57 patients (60.0%), and three days postoperatively in 23 patients (24.2%). High-output, moderate-output, and low-output CF were identified in 30 (31.6%), 30 (31.6%), and 35 (36.8%) patients, respectively.

Among the patients presenting with low-output CF, 27 cases (77.2%) were successfully resolved with traditional methods, including pressure dressing and negative-pressure drainage, with a median drainage duration of 14 days (range, 6-57 days).

Among the patients with moderate-output CF, 20 (66.7%) were successfully managed with traditional treatment alone, with a median duration of 23 days (range, 10-60 days). Five additional cases were resolved with the addition of octreotide to the traditional methods.

Among the high-output CF patients, 13 (43.3%) were effectively managed with traditional methods alone, with a median drainage duration of 30 days (range, 17-88 days). Additionally, two cases required reoperation for thoracic duct ligation after traditional methods failed, occurring at 25 days and 27 days post-initial treatment, respectively. Following reoperation, the drainage volume decreased sharply to no more than 10 mL per day.

### PAI

In total, eight patients with low-output CF, five with moderate-output CF, and 15 with high-output CF underwent PAI. The decision to proceed with PAI was based on the failure of standard conservative therapy, defined as persistent drainage of >100 mL/day after at least 5 days of appropriate management, or on clinical judgment indicating the need for more aggressive intervention to prevent prolonged hospitalization or nutritional complications.

Among the eight low-output CF patients, the median duration of prior traditional therapy was 11 days (range: 9-19), with a mean 24-hour drainage volume of 449 ± 42 mL prior to PAI. Post-PAI, the mean drainage volume declined to 21 ± 10 mL by the first day. By the second day, drainage had ceased in seven patients (87.5%; 95% CI: 47.3% to 99.7%), with one patient exhibiting approximately 5 mL of drainage for one additional day.

Among the five patients with moderate-output CF, the median drainage duration of prior treatment was 9 days (range: 7-11), and the mean 24-hour drainage volume before PAI was 644 ± 128 mL. On the first day after PAI, the 24-hour drainage volume significantly decreased to 42 ± 14 mL. By the second day, the volume was less than 20 mL in 80% of patients (95% CI: 28.4% to 99.5%), and all patients had their drainage tubes removed by the third day.

**Figure 1:**
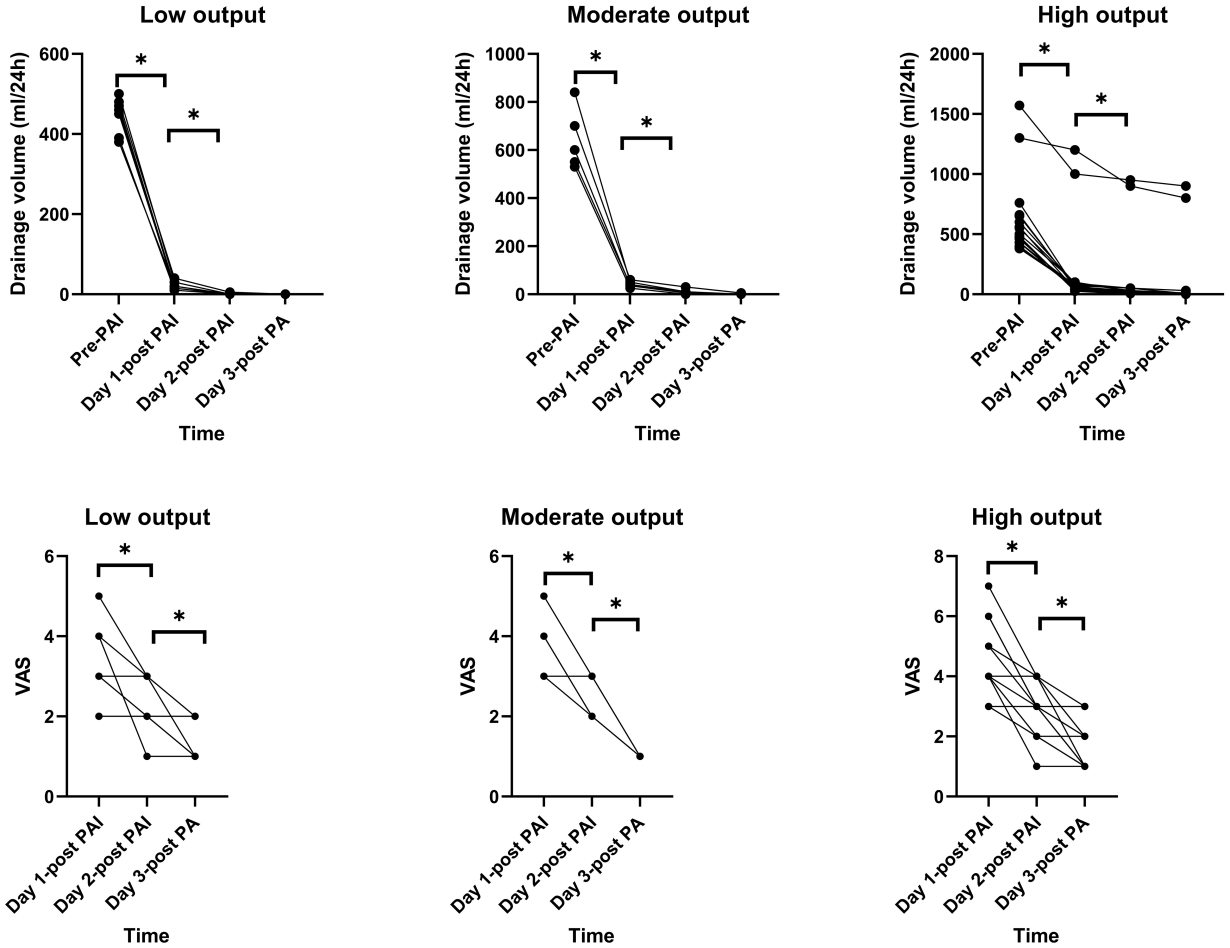
Drainage volume and pain scores following *Pseudomonas aeruginosa* injection (PAI). Mean 24-hour drainage volume in the low- (n=8), moderate- (n=5), and high-output (n=15) chyle fistula groups before and after PAI administration. Mean visual analog scale (VAS) pain scores (0-10) for each group on days 1, 2, and 3 post-PAI. Data are presented as mean ± SD. *p < 0.05 vs. previous day within group (Bonferroni-corrected).

In the cohort of 15 patients with high-output CF, two patients received PAI twice. Before PAI, the median duration of traditional treatment was 10 days (range: 5-15), with a mean drainage volume of 633 ± 348 mL. Following a single PAI injection, the mean drainage volume was 55 ± 19 mL on the first day and 24 ± 17 mL on the second day; by the third day, drainage had ceased in six patients (40.0%; 95% CI: 16.3% to 67.7%). For the two patients who underwent two PAI injections, a marked reduction in drainage volume followed each injection, and both achieved drain removal by the third day after the second PAI (**Figure 1**).

Post-PAI, 75.0% (95% CI: 34.9% to 96.8%) of patients in the low-output group and 100% in the moderate and high-output groups experienced fever on the first day. By the second day, fever had resolved in the entire low-output group, while four patients (26.7%; 95% CI: 7.8% to 55.1%) in the high-output group and one (20.0%; 95% CI: 0.5% to 71.6%) in the moderate-output group continued to exhibit fever. Notably, no fever was present in any patient by the third day. Fever was managed solely with physical cooling interventions such as ice packs; no antipyretic medications were administered (**Tables 1**–**3**).

**Table 1:** Demographic, clinical, and outcome characteristics of patients undergoing *Pseudomonas aeruginosa* injection (PAI) for chyle fistula, stratified by output level.

Characteristic	Low-output CF (n=8)	Moderate-output CF (n=5)	High-output CF (n=15)	P-Value
Demographics				
Age, years (mean ± SD)	44 ± 10	47 ± 12	45 ± 11	0.845
Male Sex, n (%)	3 (37.5%)	2 (40.0%)	6 (40.0%)	0.991
Pre-PAI Drainage				
Drainage Duration before PAI, days (median, IQR)	11 (10 - 14)	9 (8 - 10)	10 (9 - 12)	0.215
Drainage Volume before PAI, mL/24h (mean ± SD)	449 ± 42	644 ± 128	633 ± 348	0.155
Post-PAI Outcomes				
Drainage Volume, mL/24h (mean ± SD)				
Day 1	21 ± 10	42 ± 14	55 ± 19*	<0.001
Day 2	0.6 ± 1.8	11 ± 11	24 ± 17*†	<0.001
Day 3	0 ± 0	1 ± 2	5 ± 8*†	0.025
Patients with Drain Removed by Day 3, n (%)	7 (87.5%)	5 (100%)	6 (40.0%)*†	0.008
Adverse Events				
Fever on Day 1, n (%)	6 (75.0%)	5 (100%)	15 (100%)	0.105
Pain VAS Score (mean ± SD)				
Day 1	3.3 ± 1.0	3.8 ± 0.8	4.4 ± 1.3*	0.038
Day 2	2.3 ± 0.7†	2.4 ± 0.5†	2.9 ± 0.9*†	0.095
Day 3	1.4 ± 0.5†	1.0 ± 0.0†	1.6 ± 0.7†	0.083

Data are presented as mean ± standard deviation, median (interquartile range), or number (percentage). P-values were calculated using ANOVA for continuous variables and Chi-square or Fisher's exact test for categorical variables. Post-hoc pairwise comparisons were adjusted with Bonferroni correction.

* p < 0.05 vs. Low-Output group.

† p < 0.05 vs. Day 1 value within the same group.

**Table 2:** Drainage information of patients undergoing the injection of *Pseudomonas aeruginosa* (PAI).

Group	Drainage duration before PAI (day)	Drainage volume before PAI (ml/24h)	Drainage volume after PAI, day 1 (ml/24h)	Drainage volume after PAI, day 2 (ml/24h)	Drainage volume after PAI, day 3 (ml/24h)^
Low					
1	9	460	15	0	–
2	11	500	30	0	–
3	13	380	20	0	–
4	10	470	20	0	–
5	10	460	15	0	–
6	16	450	20	0	–
7	11	390	10	0	–
8	19	480	40	5	0
Moderate					
1	10	600	35	5	0
2	9	840	50	10	0
3	7	530	25	0	–
4	8	550	40	10	0
5	11	700	60	30	5
High					
1	10	760	55	20	0
2	9	560	40	5	0
3	10	430	70	10	0
4	12	390	80	50	30
5	10	660	55	25	10
6	12	430	40	20	0
7	8	470	30	10	0
8	11	460	45	15	0
9	15	400	55	30	10
10	13	380	70	50	5
11	7	480	25	0	–
12	10	550	90	50	5
13	9	650	60	30	5
14*	5	1570	1000	950	900
			100	25	0
15*	6	1300	1200	900	800
			90	30	0

* Patients of number 14 and 15 received two times of PAI.

^ -: no data.

**Table 3:** Adverse events of patients undergoing the injection of *Pseudomonas aeruginosa* (PAI).

Group	Fever^&^ after PAI			Pain after PAI		
	Day 1	Day 2	Day 3	Day 1	Day 2	Day 3
Low						
1	Yes	No	No	3	3	2
2	Yes	No	No	4	3	1
3	Yes	No	No	2	2	1
4	Yes	No	No	3	2	1
5	Yes	No	No	2	2	2
6	Yes	No	No	5	3	2
7	No	No	No	4	1	1
8	No	No	No	3	2	1
Moderate						
1	Yes	No	No	5	3	1
2	Yes	No	No	4	2	1
3	Yes	Yes	No	4	2	1
4	Yes	No	No	3	3	1
5	Yes	No	No	3	2	1
High						
1	Yes	Yes	No	3	3	2
2	Yes	No	No	5	4	1
3	Yes	Yes	No	6	3	1
4	Yes	No	No	5	3	1
5	Yes	No	No	4	2	1
6	Yes	No	No	4	4	2
7	Yes	No	No	4	3	2
8	Yes	No	No	3	3	2
9	Yes	Yes	No	3	2	2
10	Yes	No	No	7	4	3
11	Yes	No	No	3	2	1
12	Yes	No	No	5	4	2
13	Yes	Yes	No	6	3	3
14*	Yes	No	No	4	1	1
	Yes	No	No	3	3	1
15*	Yes	No	No	6	3	1
	Yes	No	No	4	2	1

* Patients of number 14 and 15 received two times of PAI.

& Fever was defined as the high body temperature exceeding 38.5°.

Following PAI, all patients reported varying degrees of pain. In the low-output group, mean VAS scores were 3.3 ± 1.0, 2.3 ± 0.7, and 1.4 ± 0.5 on the first, second, and third days, respectively, with significant differences between days (p < 0.05 for all comparisons, **Figure 1**). In the moderate-output group, the mean VAS score was 2.4 ± 0.5 on the second day, which was significantly lower than on the first day but higher than on the third day. In the high-output group, mean VAS scores were 4.4 ± 1.3, 2.9 ± 0.9, and 1.6 ± 0.7 on the first, second, and third days, respectively, with significant differences between time points (p < 0.05 for all comparisons, **Figure 1**). Pain was managed with oral non-opioid analgesics as needed, and all patients achieved adequate pain control (**Tables 1**-**3**).

### Proposed CF treatment flowchart

**Figure 2:**
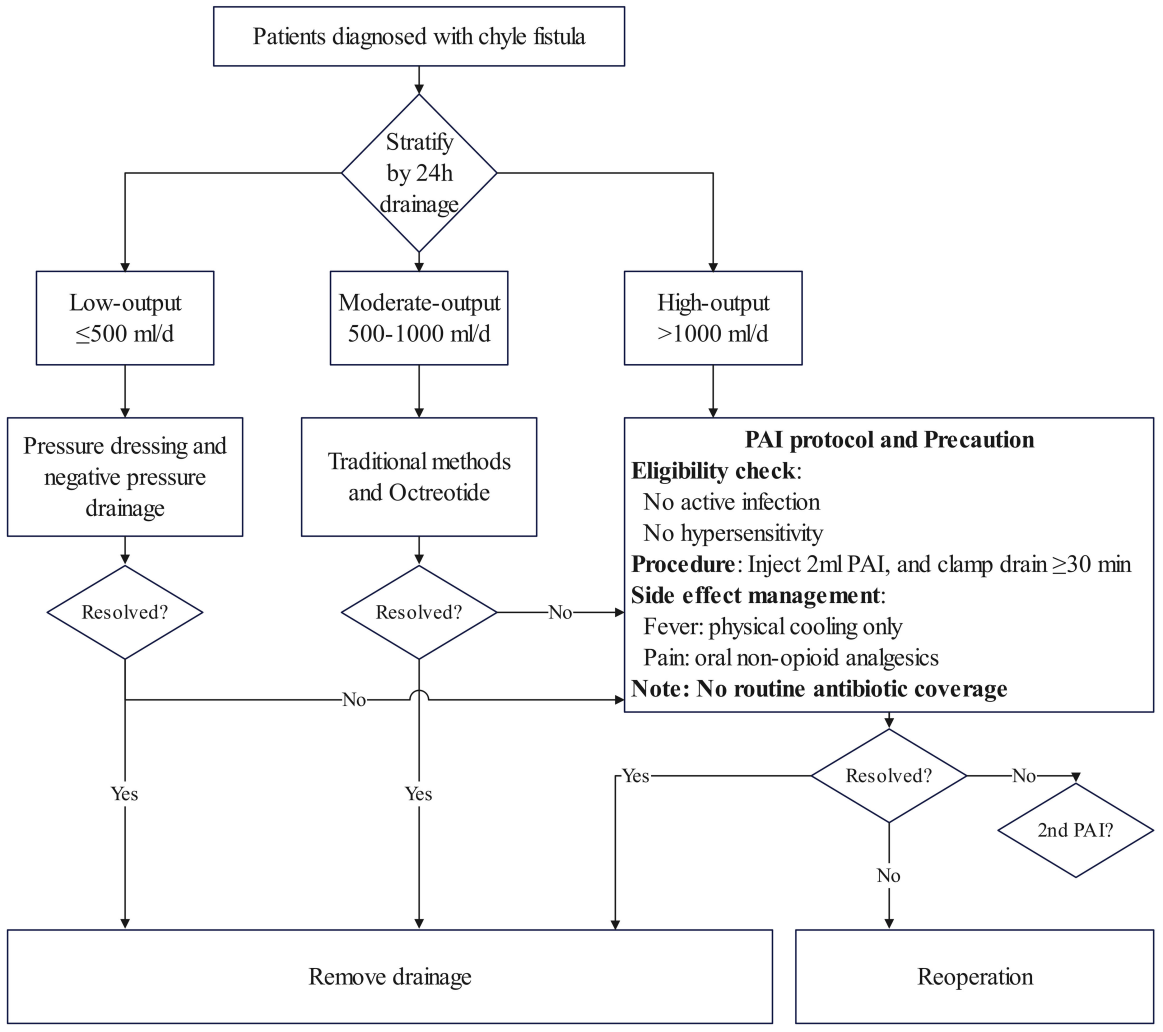
Proposed tiered management algorithm for chyle fistula (CF). The application of *Pseudomonas aeruginosa* injection (PAI) is recommended for persistent low-output CF, persistent moderate-output CF, and as a primary intervention for high-output CF. Crucially, PAI should only be considered for immunocompetent patients without active systemic infection. The PAI preparation is inactivated and does not require concomitant antibiotics. Post-procedural fever should be managed with physical cooling to avoid potential interference with the immunostimulatory mechanism, while pain can be controlled with standard oral analgesics. Reoperation remains the final option after failed conservative and PAI management.

To summarize the optimal management of CF, we propose a treatment algorithm (**Figure 2**). For low-output CF, initial management with a combination of pressure dressing and negative-pressure drainage is recommended, with PAI indicated if this regimen fails. For moderate-output CF, first-line therapy should consist of traditional methods combined with octreotide, transitioning to PAI if there is an inadequate response. For high-output CF, PAI is recommended as the primary intervention. In all instances, reoperation is reserved as the final therapeutic option following failed PAI treatment.

## Discussion

Our principal finding revealed that, on one hand, PAI effectively addressed CF cases that were previously unresolved by other non-surgical interventions, while incurring only minor complications associated with thyroid surgery. On the other hand, it is evident that CF necessitates a structured, tiered management approach based on varying output levels. We aspire that this research introduces a groundbreaking non-surgical treatment for CF, characterized by high efficacy and a limited incidence of adverse events.

CF, though typically rare, poses a potentially life-threatening risk, with an incidence of approximately 1% observed during neck dissection in thyroid surgery (2). The treatment of refractory CF can be both time-consuming and financially burdensome. PAI has been shown to elicit localized aseptic inflammation, fostering adhesion between the skin and muscle wounds and expediting the closure of small lymphatic vessels, thereby facilitating wound healing. Its application has been documented in various clinical scenarios, including malignant pleural effusion, pericardial effusion, and persistent effusion following breast cancer surgery (7). Notably, a medical team at West China Hospital was the first to propose PAI as a treatment for CF in four patients with thyroid cancer (13). In their study, the four patients reported 24-hour drainage volumes of 200, 350, 540, and 810 mL prior to injection, with two patients experiencing CF and drainage durations of 7 and 15 days, respectively; the remaining two patients dealt with simple lymphatic leakage and had drainage times of 13 and 14 days, respectively. Remarkably, one day post-injection, the 24-hour drainage volumes decreased to 20, 45, 120, and 255 mL, respectively, and by the fourth day, all four patients successfully had their drainage tubes removed. While all patients experienced varying degrees of fever following PAI, their body temperatures normalized with physical cooling interventions. Localized pain was reported among all patients, with three managing to tolerate the discomfort and one patient experiencing severe pain that was alleviated with oral meloxicam. This study elucidated the role of PAI in managing CF subsequent to thyroid surgery. Wei et al. (11) retrospectively compiled their experience with 18 patients suffering from CF, noting that after PAI, chyle output exhibited significant reduction and ceased within 3 to 5 days in 17 patients. The sole patient who did not respond to the initial application underwent a second PAI treatment, after which chylous leakage ceased within three days, enabling subsequent removal of the drainage tube. Regarding complications, mild fever was observed in nine patients, moderate fever in four, and severe fever in five. All patients experienced some degree of neck pain. Notably, follow-up over a median duration of three years revealed no long-term adverse effects. Furthermore, the research team designed a randomized, parallel-group, placebo-controlled trial involving 200 patients, who were assigned to either the PAI group or the control group. The results indicated that patients in the PAI group experienced a significant reduction in macroscopic CF compared to the control group (0% vs. 6%). Additionally, the PAI group required fewer days for drainage tube removal, produced a lesser volume of drainage fluid, experienced shorter postoperative hospital stays, and had a reduced red blood cell count in the drainage fluid. Importantly, no severe side effects related to PAI spray, including fever, pain, or pleural effusion, were documented, and PAI spraying did not adversely impact the postoperative recurrence of thyroid cancer. Subsequent studies from the same group further confirmed the safety and efficacy of PAI in decreasing drainage volume and shortening hospital stays after lateral neck dissection (8, 10, 12).

However, validation of these findings by other researchers has been rare, as we are only aware of two available papers for analysis. The first was a case report documenting successful resolution of moderate-output CF via PAI (14), while the second was an original research study involving 69 thyroid cancer patients with CF (15); it compared outcomes of 37 patients who underwent conventional negative pressure drainage via bilateral tube placement with those treated by PAI. The observational group demonstrated significantly shorter drainage times compared to the control group. Some cases in the observation group experienced adverse reactions such as local fever and chills within two weeks post-surgery, with body temperatures returning to normal following physical cooling. Although the overall incidence of adverse reactions was higher in the observation group compared to the control group (12.50% vs. 8.11%), the difference did not achieve statistical significance. In our current study, we likewise noted the successful resolution of CF via PAI after the failure of prior non-surgical management techniques, with only minor complications observed. Collectively, these findings underscore the high efficacy of PAI in managing CF, with the underlying mechanisms being attributable to the biological characteristics of PAI, which are well recognized in existing literature.

The optimal management of CF has yet to achieve broad consensus, with treatment decisions primarily guided by the surgeon’s expertise and personal preference. Generally, the drainage output serves as the most reliable indicator for selecting a treatment modality, with conservative management typically employed as the initial approach. Santaolalla et al. (16) reported on four CF cases, noting complete resolution in three patients through traditional methods, although details on the drainage volumes were not provided. Our current study observed that nearly 80% of low-output CF cases could be effectively managed with traditional approaches alone; however, this success rate diminished to 66.7% for moderate-output cases and further declined to 43.3% for high-output cases, with both latter categories experiencing drainage durations exceeding two weeks. This finding corroborates the conclusions of previous authors (17), who suggested that a two-week conservative treatment period should be observed prior to considering alternative therapies.

Octreotide has been recognized for its high efficacy in managing CF (18). Swanson et al. (4) reported that among 12 patients who received octreotide combined with negative pressure drainage or pressure dressing, eight cases resolved within one week; notably, seven patients presented with low-output CF, three with moderate-output CF, and one with high-output CF. Chan et al. (19) also demonstrated the effectiveness of octreotide and peripheral total parenteral nutrition in treating CF in a cohort of ten patients, achieving control of chyle leaks in eight (80%) of the cases, while two patients ultimately required surgical intervention. Interestingly, no factors emerged as significant predictors for successful conservative management of CF, with one patient presenting with a drainage volume of 1,800 mL/day achieving resolution without surgical intervention. In our investigation, successful treatment was achieved in 20 (80%) of the 25 patients administered octreotide, with all failures occurring in cases that did not incorporate PAI. While octreotide is a valuable non-surgical tool, its efficacy may be limited in high-output or persistent leaks, and it often requires several days to take effect.

Surgical re-intervention, such as thoracic duct ligation or embolization, remains the final recourse for CF management, consensus surrounding the optimal timing and procedural steps for managing the leakage has yet to be established (20, 21). As indicated in a prior review (5), if leakage occurs postoperatively, management strategies should be guided by the daily output of the leakage; for high output levels (1-4 L/day) or prolonged low output following 7 to 14 days of conservative management, exploratory surgery is deemed the principal treatment to arrest the leakage. Alternatively, thoracic ductal embolization or thoracoscopic thoracic ductal ligation represents a minimally invasive option that aligns with our findings. In instances where the output exceeds 4 L/day or if the aforementioned interventions fail, employing a pectoralis major myocutaneous flap has been regarded as a viable therapeutic option to rescue patients from potentially life-threatening complications. While we concur with this strategic framework, our current study, in conjunction with other research, highlights the considerable efficacy of CF control that exists independently of the 24-hour drainage volume. Therefore, prior to considering reoperation, we strongly advocate for the use of PAI as a less invasive and highly effective intermediary step.

It is important to acknowledge that the use of a preparation derived from *Pseudomonas aeruginosa*, even in an inactivated form, may raise biosafety and regulatory concerns in some international settings. The PAI used in our study is a non-viable, inactivated suspension approved by the National Medical Products Administration of China for malignant effusions and used off-label for CF in our institution. Its safety profile in this and previous studies has been favorable, with no evidence of systemic infection or long-term sequelae. However, regulatory approval and clinical acceptance of bacterial-derived therapies vary globally, and further multi-center, international studies may be needed to establish PAI as a standard treatment option for CF in diverse healthcare systems.

Furthermore, from a health-economic perspective, PAI presents a potentially cost-effective alternative. While a formal cost-analysis was beyond the scope of this study, the significant reduction in drainage duration and time to drain removal observed with PAI likely translates into substantial cost savings. Prolonged conservative management, particularly when involving parenteral nutrition and extended hospitalization, incurs considerable expenses related to nutritional support, nursing care, and inpatient bed days (4, 18). Although the unit cost of the PAI preparation itself is a factor, its ability to rapidly resolve CF and facilitate early discharge is expected to offset these initial costs by reducing the overall length of stay and consumption of hospital resources. In contrast, surgical re-intervention, such as thoracic duct ligation, involves the significant costs of a second operation, operating room time, and anesthesia. Therefore, the integration of PAI into a tiered management protocol may not only improve patient outcomes but also alleviate the economic burden associated with protracted CF management.

In our study, post-PAI fever was managed exclusively with physical cooling methods, and systemic antipyretics were deliberately avoided. This approach was based on the understanding that the efficacy of PAI is likely mediated by the induction of a localized inflammatory and immunostimulatory response (6, 11). As fever is a systemic manifestation of this inflammatory cascade, we hypothesized that suppressing it with anti-inflammatory drugs could potentially attenuate the desired therapeutic effect. The successful resolution of CF in our cohort without the use of antipyretics supports the feasibility of this management strategy. However, whether the use of antipyretics would indeed reduce the efficacy of PAI remains a pertinent question for future prospective studies to investigate.

Regarding the suitability of PAI for different patient classes, our study did not identify any specific prerequisites related to immune cell counts or nutritional status for treatment success. The regimen was effectively applied across a spectrum of patients, including those with elevated BMI. Based on our protocol and experience, the primary contraindication for PAI is a compromised immune system, as the immunostimulatory mechanism of action is central to its efficacy and safety in an inactivated form. Therefore, we would not recommend its use in patients with significant immunodeficiencies. Similarly, PAI should be avoided in individuals with active systemic infection or known hypersensitivity to the components of the injection. For the typical patient undergoing major neck surgery for thyroid cancer, who is generally immunocompetent, PAI appears to be a suitable and effective treatment option for refractory chyle fistula. Future studies with larger cohorts could further stratify outcomes based on specific comorbidities.

An important consideration is whether this PAI regimen is applicable to CF following neck dissection for head and neck squamous cell carcinoma (HNSCC), where CF incidence is higher and management is particularly time-sensitive due to the need for timely adjuvant radiotherapy (22). While our current study focused specifically on thyroid cancer patients, the pathophysiological mechanism of chyle leakage is common to both entities. The biological action of PAI, which promotes localized inflammation and tissue adhesion to seal lymphatic leaks, should theoretically be equally effective regardless of the underlying malignancy (23). Although not systematically analyzed in this series, our center has successfully applied the same PAI protocol in selected HNSCC patients with refractory CF when rapid resolution was required to avoid delays in adjuvant radiotherapy. Based on this experience and the shared mechanism of lymphatic injury, we believe PAI represents a promising intervention for CF in HNSCC patients. However, this warrants formal validation through prospective studies specifically designed for the HNSCC population, particularly considering their distinct comorbidities and frequent need for multimodal therapy.

The limitations of the current study must be duly acknowledged. Firstly, this investigation was retrospective in nature, inherently introducing a potential for selection bias. Secondly, our sample size was relatively modest, which may have diminished our statistical power. Thirdly, cases of CF exceeding 2000 mL per day were not included in this study, indicating the necessity for further research to verify the efficacy of PAI in such instances.

In conclusion, PAI has demonstrated effectiveness in addressing CF cases that were inadequately managed by prior non-surgical methods, while incurring only minor complications associated with thyroid surgery. It is evident that CF requires a structured, tiered management approach that accommodates varying levels of severity.

## Data Availability

The raw data supporting the conclusions of this article will be made available by the authors, without undue reservation.
